# Challenges in implementing and assessing outcomes of school start time change in the UK: experience of the Oxford Teensleep study

**DOI:** 10.1016/j.sleep.2018.10.021

**Published:** 2019-08

**Authors:** Gaby Illingworth, Rachel Sharman, Adam Jowett, Christopher-James Harvey, Russell G. Foster, Colin A. Espie

**Affiliations:** a Sleep and Circadian Neuroscience Institute, Nuffield Department of Clinical Neurosciences, University of Oxford, UK; b Department of Psychology, Royal Holloway, University of London, UK

**Keywords:** Adolescent sleep, School start time, Sleep education, Research design, Randomised controlled trial

## Abstract

**Objective:**

Later school start times for adolescents have been implemented in the US and associated benefits found, although no randomised controlled trials (RCT) have been undertaken. The objective of this study was to evaluate the impact of two school interventions in the UK, a delayed start time and a sleep education programme, on students’ academic performance, sleep outcomes and health-related quality of life.

**Methods:**

The study had an RCT design to enable an investigation into the differential effects of two interventions or a combination of both: schools were to delay their start time to 10:00am and/or provide a classroom-based sleep education programme. The recruitment target was 100 state (non-fee-paying) secondary schools. Participants were to be students in Year 10/11 (14–16-year-olds).

**Results:**

Despite much media coverage, only two schools volunteered to take part in the RCT. The main challenges faced in recruitment fell under three categories: research design, school, and project-specific issues. The delayed start time and prospect of randomisation to this intervention were the overwhelming reasons cited for not taking part. Facilitators and barriers to research were identified. Recommendations include carrying out a feasibility study prior to a main trial, allowing adequate time for recruitment, involving stakeholders throughout the decision-making process, incorporating independent (fee-paying) schools in recruitment, focusing on students not taking important examinations or involving an older year group with greater independence.

**Conclusion:**

The Teensleep study provides supporting evidence that evaluating the effects of a change in school start times through an RCT is unfeasible in the UK.

## Highlights

• Study suggests it is not feasible to recruit schools to a randomised controlled trial involving delayed start times in UK.
• Delayed start time and randomisation were primary reasons cited for not taking part.
• Recommendations concern feasibility study, timing, stakeholders, target schools and year groups.
• Highlights the need for a pragmatic approach to evaluating school start times.

## 1 Introduction

During adolescence, our circadian timing system delays and homeostatic sleep pressure accumulates more slowly during the day resulting in a preference to fall asleep and wake later [1], [2], [3]. This biological pre-disposition for delayed sleep may then be exacerbated by environmental, behavioural/lifestyle and psychosocial factors [4], [5], [6]. Societal expectations also play a part, in particular school start times that require students to get up early. These contribute not only to sleep loss by curtailing the time available for sleep but also, if adolescents are forced to waken before they would naturally in their circadian cycle, to a state of circadian misalignment and ‘social jetlag’ [7]. It is little surprise then that a global pattern of chronic sleep-deprivation in this age group has been documented in recent years [8], [9], [10], as well as an increasing delay in bedtimes and a growing mismatch between sleep on school days and sleep at the weekend [11]. Also of concern, some evidence suggests that adolescent sleep duration has been declining over the last century [12], [13].

Given that insufficient sleep increases the risk for impairments to physical health, mental health and behaviour [14], [15], [16], researchers, working in conjunction with educators, are looking at two alternative approaches to address this issue. Schools are central to this process because of their ability to work at an organisational level to help improve adolescent sleep. Sleep is likely to be of particular interest to educational institutions due to the links between cognitive and academic performance and sleep quantity and quality [17], [18], [19]. Delaying school start times and sleep education programmes within the classroom are the interventions of choice, focusing on potentially modifiable intrinsic and extrinsic factors affecting sleep.

Later school start times, proposed to better align with the adolescent phase delay and sleep patterns, is an intervention that aims to address biological factors affecting sleep. Allowing adolescents to sleep to their chronotype and begin the day later can increase the potential for more sleep each school night as well as letting students be in school during the hours when the majority feel alert and ready to learn. The most intensive and prominent campaign for later school start times has taken place in the US. Compelling evidence has demonstrated that delaying school start times associates with positive outcomes such as increased sleep duration, reduced daytime sleepiness and improved mood [20], [21], with some evidence supporting improvements in academic performance [22], [23]. Notably, early school starts for middle and high schools are defined in the US as before 8:30am by major medical organisations, for example, American Academy of Pediatrics [24] and American Academy of Sleep Medicine [25], whereas most schools in the UK begin at around 9:00am. An urban state school, in an area of England with achievement lower than the national average, delayed their start time from 8:50am to 10:00am for two years and found that the delay associated with reduced rates of absence due to illness and improved academic performance [26]. However, the study was observational so no sleep data were collected and the earlier start time was reinstated after two years following a change in local education administrators.

Sleep education, on the other hand, is an intervention that aims to address behavioural/lifestyle and psychosocial factors that may negatively impact sleep and contribute to an evening chronotype incompatible with early awakenings. The sleep education programmes implemented so far vary in terms of theoretical background but in general aim to increase adolescents’ knowledge about sleep and/or improve sleep behaviours [27], [28], [29]. There is some evidence that changes in sleep and sleep behaviour follow a sleep education programme [30], [31], [32], [33]. Promisingly, although the study included children (7–11 years) rather than adolescents, Gruber and colleagues found that a sleep education programme associated with an improvement in objective measures of sleep and academic performance in mathematics and English [34].

Studies investigating delayed start times and sleep education in schools have differed from each other regarding the use of their chosen research design. Evaluations of school start time change have predominantly used cross-sectional designs comparing students at different schools with earlier and later start times, or the same schools before and after a delay but not necessarily the same students [23], [35], [36], [37] rather than pre-post, prospective designs [20], [38], [39] or both cross-sectional and prospective designs within one study [21]. A meta-analysis of later school start time research concluded that more prospective studies were needed [40]. Moreover, school start time studies have not used a randomised controlled trial (RCT) design to assess outcomes and this has been cited as a methodological weakness [40]. A systematic review called for a randomised design in future studies [41], while another review called for trials with controls [42]. To our knowledge, the only study that has investigated the impact of delayed start times for adolescents using an RCT paradigm, utilised a within-school design where one class experienced delayed start times for one week and one class acted as a control group [43]. In contrast, researchers investigating the impact of sleep education have utilised an RCT design in recent years [30], [31], [33], [44].

To date, these two approaches have only been trialled in isolation and not in combination. Although a healthy school start time has been proposed to be a necessity, as a single strategy it may not be enough to encourage sleep health and may require an additional strategic focus on the individual, for example their sleep hygiene behaviours [45]. This article discusses the challenges faced by the Teensleep study to address this gap in the literature. The purpose was to investigate adolescent sleep and academic outcomes using a prospective research design in schools utilising either a single intervention, a combination of two interventions, or ‘carrying on as usual’, and to do so in conjunction with a random allocation to these conditions.

The Teensleep study was one of six education and neuroscience projects funded by the Wellcome Trust and the Education Endowment Foundation (EEF) in the UK. The EEF is an independent charity established to improve the educational attainment of 3–18 year olds, particularly those from disadvantaged backgrounds. An EEF research protocol has the following stipulations: an independent evaluator is involved in addition to the study team; only state (non-fee-paying) schools can participate; an RCT is the predominant research design. The primary aim was to assess the impact on students’ academic outcomes through introducing a later school day and/or sleep education. Academic outcomes were to be measured using General Certificate of Secondary Education (GCSE) grades: the main academic qualifications taken at the approximate age of 16 years in England and Wales. The secondary aims were to assess the impact of the interventions on sleep outcomes and health-related quality of life.

## 2 Methods

### 2.1 Design

The Teensleep study had a factorial cluster RCT design to enable an investigation into the differential effects of the two interventions: delayed school start time and sleep education. If randomised to this condition, schools were to be given a year to make arrangements before they were required to implement the change in their start times the subsequent academic year. The study received ethical approval from the Universities of Oxford, Durham and York.

### 2.2 Participants

All students in Year 10 and Year 11 (14–16-year-olds) in participating schools were to be included in 2016–2017, the first year of the study. Year 10 students moving into Year 11 were to be included in 2017–2018, the second year of the study. Due to the interest in academic outcomes, this age group was chosen to take part as these are the two academic years that focus on GCSEs.

### 2.3 Interventions

Schools had the choice of changing the start time only for the study participant year group (Year 11) or they could choose to delay the whole school to 10:00am. The average sleep duration of a UK adolescent was not available due to the paucity of sleep monitoring undertaken with this age group in the general population. It was not known therefore how much more sleep, if any, was needed by students in order to meet the recommended guidelines of 8–10 h for 14–17 year-olds provided by the National Sleep Foundation [46]. Instead, the adolescent shift to an evening chronotype informed the decision to set an alternative 10:00am school start time, an average delay of approximately one hour, in order to increase the likelihood that students would feel fully awake and prepared for learning in the first lesson of the day. This was of particular importance given the focus on academic performance. Sleep education was to be provided as a flexible package in PSHE (Personal, Social, Health and Economic) lessons. This teacher-led, educational intervention was to be evaluated in a number of schools in the year before randomisation took place to enable student and teacher feedback to be given and any subsequent amendments to be made for the main trial.

### 2.4 Assessment

Self-report measures of sleep and health-related quality of life were to be collected from each year group three times (beginning, middle and end of academic year) in Year 1 and Year 2. In addition, a subgroup of students in each school was to take part in sleep monitoring involving actigraphy and sleep diaries. Academic achievement was to be assessed through GCSE grades at the end of both academic years.

### 2.5 Recruitment and randomisation

**Fig. 1:**
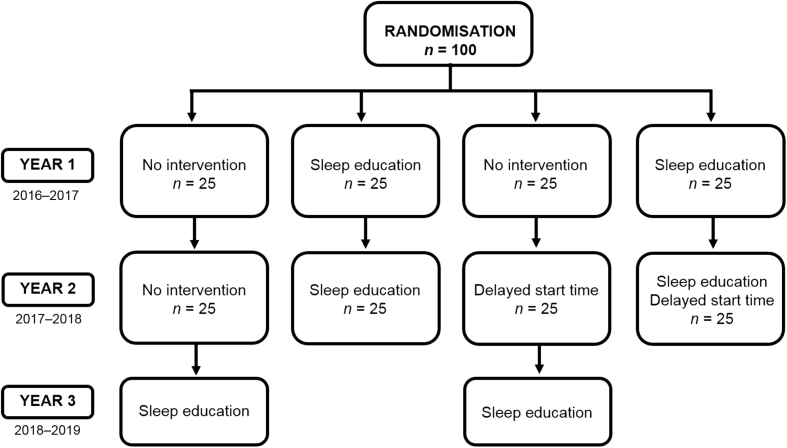
Overview of the RCT design including recruitment targets and timeline.

The recruitment target was set at 100 state secondary schools across England and Wales by the study evaluators and the EEF. Schools were to be randomised to one of four conditions by the study evaluators using minimisation (random dynamic allocation) to ensure that each condition contained schools balanced on important covariates, for example, school's cohort (year group) size, indication of level of deprivation measured by free school meals, and academic attainment in the year prior to recruitment. The RCT design and recruitment targets are summarised in Fig. 1. Randomisation was to occur once all schools had signed up and provided necessary baseline data, with 25 schools randomised to each condition. A financial contribution of £1000 was to be given to each school to acknowledge the time and resources required to take part in the study. Schools not involved in the sleep education intervention were to be given access to the programme after the end of the study.

#### 2.5.1 Recruitment strategies

The Teensleep team began recruitment for the study in 2015, using numerous techniques to raise awareness of the study with head teachers, teachers, parents and students and to encourage them to want to take part. These are outlined in brief below to highlight the reach and breadth of these strategies:

• Promotional online films

Films to explain study rationale and research design to schools were available on the study webpage (www.teensleep.org.uk) and Oxford Sparks website (http://bit.ly/OSSleep), the university website dedicated to public engagement and science outreach.

• Email service

An introductory email was sent using an educational marketing agency's database of contacts to 3985 state secondary schools in England and Wales.

• Direct contact

400 schools and other potentially useful resources were contacted directly.

• Advertising

Advertisement in MyAcademy Magazine was sent to 4989 academies (type of UK school) and approximately 20,000 people.

• Social media

Facebook and Twitter, websites, posters and newsletters.

• Recruitment events

Hosted four recruitment events across England and presented at others.

• Advisory group

High-profile individuals agreed to help attract schools and work with the media.

## 3 Results: how did we do?

### 3.1 Media coverage

There was much media interest in the study, predominantly because of the topic of school start time change. Teensleep was featured by major newspapers, radio stations and television broadcasters, therefore awareness of the study was high.

### 3.2 Recruitment

By early 2016, 27 schools had shown an interest in the study but did not confirm they would take part and 35 schools had shown an interest but subsequently declined to take part. A total of two schools had confirmed they wanted to take part in the RCT. One of these schools was a new academy school that had not yet opened and the other was a boarding school. Therefore, at this point and given these numbers, the continuation of Teensleep as an RCT was decided not to be feasible.

### 3.3 The challenges

During the recruitment process, schools gave feedback to the study team on why they were interested in the study and why they ultimately would not participate. The main challenges faced in recruitment to the RCT are detailed below. These fall under three categories: research design, school, and project-specific issues. The focus here is on the most-often cited barriers and predominant concerns but this is not to say that other issues were not raised within these categories.

#### 3.3.1 Research design issues

##### 3.3.1.1 Delayed start time and prospect of randomisation

The overwhelming message was that schools did not feel able to delay their start times, therefore the randomisation aspect of the research design (ie, the RCT paradigm) meant that they were not able to sign up to the study. Schools reported that if the study design changed, and they could opt out of this particular intervention or self-select an intervention, they would be able to take part. In contrast, the prospect of providing sleep education or being in the control group were considered to be attractive possibilities.

##### 3.3.1.2 Decision-making and timing

**Fig. 2:**
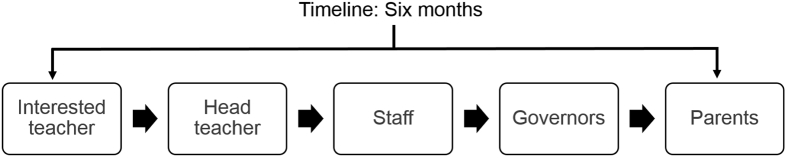
Flowchart of a typical decision-making process for a school to agree to change their start time.

The decision-making process to get the required support from all stakeholders needed to agree to a delayed start time emerged as a potentially lengthy process. It was reported by a school staff member that it would be likely to take approximately six months to get sign off from all stakeholders at their institution. The stakeholders and order of consultation and agreement are presented in Fig. 2. After this involved and time-consuming process was completed, and if the school had agreed that they were amenable to changing their start time, the study design meant that this school might then be randomised to a different condition. As a result, schools might face a difficult situation in that stakeholders wanted to change their start time and yet could not do so if they were to remain in the study.

#### 3.3.2 School issues

##### 3.3.2.1 Academic performance

Schools reported that there were exceptional expectations for academic performance. The study's focus on and inclusion of Year 11 students proved problematic as this is the year group that takes GCSEs. Schools reported it would not be possible to delay the start of the school day for these students and that parents would not appreciate this in the lead-up to exams. Furthermore, even if the schools did delay their start time, it was pointed out that GCSE morning examination times are set nationally and begin at 9:00am, and so a delay might then be counter-productive for students not accustomed to starting the day at this time. Ofsted (Office for Standards in Education, Children's Services and Skills) was also cited as an impediment to taking part. This non-ministerial department of the UK government carries out school inspections and evaluates performance and as such, a school's focus might be on maintaining or improving standards to meet set requirements. GCSE results contribute to Ofsted's assessment of school performance.

##### 3.3.2.2 Examination changes

GCSEs, the main outcome measure, were also in the process of changing during the projected timeline of the study. Schools faced the additional challenge of dealing with new and more demanding content, a different structure of assessment that would be mainly by examination, and a new grading system. The process was to begin in England with just three subjects changing in September 2015, with a staggered change of all subjects by September 2017. This led to much media coverage and concern that the changes were not only complicated but also more alarmist speculation that these were possibly going to be detrimental to the students in the first wave of changes, due to varying methods of assessment and multiple grading systems.

##### 3.3.2.3 Extremely limited time and resources available for research

Schools emerged as organisations facing many pressures on their time and on their financial resources. A delayed start time was viewed as a major change that was not within their power to implement. Instead, their focus was on current priorities with budget cuts compounding this position.

#### 3.3.3 Project-specific issues

##### 3.3.3.1 Transport to and from schools is a main barrier

Schools reported that they would not be able to delay the start of the school day because of transport, with the majority of students travelling to school via bus (general public transport) or coach (chartered by school). This was a complicated logistical issue that was often considered by schools to be outside of their control and which would require a great deal of negotiation to change. A delayed start was cited as very difficult to arrange because of students travelling to school by coach, with little flexibility in the transport scheduling available. A rural intake of students meant that issues with transport could make a delayed start unfeasible.

##### 3.3.3.2 Concern about change for teachers and parents

Head teachers viewed changing the school day as a potential burden for teachers and parents. Telling staff that their conditions of service would change was not an attractive prospect for them. The impact on families was also thought to be relevant with the concern that a large strain would be placed on families' goodwill and on their management. Family arrangements, for all involved, would have to change. A later start time would affect teachers’ families, as well as parents who had to be at work by 9:00am, and those who were involved in taking children to school. One school reported that this would be an issue for parents who relied upon older siblings to accompany their younger siblings to school. Schools also reported concerns about the impact of needing to delay after-school clubs and inter-school competitions.

### 3.4 Was sleep and sleep education of interest to schools?

It is important to note that the topic of sleep and sleep education was of great interest to schools, and that overwhelmingly it was the delayed start time and the prospect of randomisation to this intervention which meant that schools did not sign up to take part in the RCT. In stark contrast to the possibility of changing school start times, sleep education was perceived to be a very attractive option for schools. Following on from the lack of recruitment success for an RCT including a delayed start time, it was agreed in a joint decision by the funders and the study team that the focus of Teensleep from Spring 2016 onwards would be solely on evaluating the effects of sleep education in schools. The results of that study will be reported in other publications.

## 4 Discussion: what we have learned

There are many potential reasons for resistance to change, including fearing an alteration to established routines and wanting to maintain the status quo. It is highly likely that this influenced the decision of UK schools to not take part in the Teensleep RCT as similar reasoning has been reported on the decision-making of US schools concerning whether or not to change start times [23]. There is no denying that changing the start of a school day would affect a variety of stakeholders and have consequences for the everyday routine and scheduling of those working in schools, families, students and the wider community. However, being prepared for resistance to change and anticipating, and responding to, potential objections can be useful. For example, the perception of teachers that they would be worse off and would work longer hours with a change in the school day could be counteracted by a positive statement from teachers at a school that had delayed. Providing support for this suggestion, while negotiating the process of change, high school principals have successfully used information from US districts that have already delayed school start times [47]. We have detailed below what we learnt during the course of recruitment for the Teensleep RCT, identifying facilitators and barriers to changing the school day, as well as some recommendations for an alternative approach for evaluating delayed school start times.

### 4.1 RCT design with delayed start times may not be realistic

An RCT including delayed start times was not shown to be a viable research design in the UK. That is not to say that schools would not agree to delay their start times given sufficiently enticing reasons and favourable conditions. However, a research design including the prospect of randomisation to this intervention would also stop an evaluation of other potentially useful interventions from taking place. The response of schools to the Teensleep study does provide supporting evidence for the view that conducting an RCT on school start times is unfeasible [45]. Although changing school start times in the UK may well prove to be an uphill struggle, various potentially beneficial strategies to increase the likelihood of success have emerged from the experience of the Teensleep study.

### 4.2 Feasibility study

A feasibility study looking at the delivery of the sleep education programme within schools was written into the RCT protocol, but given the timelines of the study and the time required for a school to be able to delay its start time, this had not been planned for the delayed start time intervention. However, a feasibility study run with a few schools may have provided information to help other schools agree to delay their start time in a larger trial, and produced solutions to any logistical issues which had been voiced as potential impediments.

#### 4.2.1 How change was implemented/potential obstacles addressed

It may have been beneficial to gather stories from a small number of schools about how they instituted the process of changing their start times as well as how they overcame any barriers. These examples could have helped address potential concerns and knowing that other, possibly similar schools, had successfully delayed their start times could be reassuring and encouraging enough for another school to begin to consider making this change. This may also provide evidence that some potential concerns raised by schools do not arise in actuality, mirroring what was found by Owens and colleagues in an examination of the process of school start time change in the US [48].

#### 4.2.2 The financial cost of making changes

Although the financial cost of changing a school start time is likely to be particular to each individual school, details of how much it had actually cost other schools to make this change, for example alterations to transportation and staff contracts, could be used as ballpark estimates for schools considering making this decision. Additionally, it could inform researchers of appropriate financial reimbursements for research schools.

#### 4.2.3 Effects on sleep, wellbeing and academic outcomes

Potential benefits, using concrete rather than hypothetical examples, which may have arisen in association with delaying school start times could have been useful in recruiting to a main trial. For example, teacher reports of possible improvements in the classroom following a change in start time, perhaps reduced levels of sleepiness, improved mood and increased attention, could provide helpful feedback from fellow professionals in relation to these key outcomes to encourage other teachers to take part later on. Supporting evidence for the benefits of later school start times from the US was provided during the recruitment process of the RCT but given that the start times of delayed schools were earlier than current UK start times, these findings were not as helpful or relevant as they may have been.

#### 4.2.4 Attitudes to school start time change

Positive stories and feedback from teachers, parents and students from their personal experiences of a different start time could then have been provided in the form of easily accessible vignettes for other schools considering delaying. For example, the impact on families could be communicated, perhaps addressing fears that working parents would be adversely affected or that travel to and from school might actually be easier because it happens outside rush hour.

### 4.3 Allow adequate time for recruitment

It is doubtful that a fast-track approach to recruitment can be successful. The process of agreeing to a change in the school day is a lengthy and complicated process that is unlikely to be rushed through. It is clear that allowing a year to recruit 100 schools was not sufficient, however it is not known how many schools could have been recruited given a less demanding research design.

### 4.4 Top-down approach problematic

The recruitment strategy of focusing on the head of a school to gain their interest and to succeed in getting them to agree to take part in the study was only the first step in a complicated process. It became clear that even if a head teacher were open to considering delaying the start of the school day, actually making this decision would involve the buy-in and approval of many stakeholders: teachers, support staff, governors, parents/caregivers, and students themselves. Although having an enthused individual on board is likely to help lead the process of change within a school, this was not a decision that would simply filter down from ‘the top’. An alternative approach would be to involve stakeholders throughout the decision-making process, for example Parent Teacher Associations and school governing boards, acknowledging that their agreement and participation would be essential for such a change to take place. Being part of the consultation process may reduce resistance and allow group and individual concerns to be addressed and possible solutions to any issues to be found. Above all, although the Teensleep study generated a great deal of media coverage, awareness of the potential benefits to the health, wellbeing and academic performance of students still needed to be communicated to a variety of stakeholders. Public discussions involving the whole community could be useful in facilitating this process, although the timescale for a positive resolution may well have been outside the scope of the Teensleep study. For example, a review of school start time research cited that changing the start time ‘can take years to accomplish’ [42].

### 4.5 School eligibility

The Teensleep study could only focus on recruiting state schools due to funder requirements. The vast majority of schools in the UK are state schools while only approximately seven per cent of schoolchildren are privately educated, so considering numbers alone this would not appear to be an issue. However, there is an argument that private/independent schools may have more freedom to make decisions over their start time and greater potential to gain the support of interested parents. In addition, these schools also have the ability to dictate their own curriculum and the freedom that entails. Widening the net to include fee-paying schools, targeting day and boarding schools, may have aided recruitment.

### 4.6 Academic pressures/year group

The age and academic pressures facing participants asked to delay their start time may be instrumental to the success of gaining stakeholder approval. This study's focus on participants facing key examinations was an additional complication. Schools were reticent to include students in an important examination year and so the study's focus on the two year groups studying for GCSEs proved to be a barrier. Without this outcome measure, it may have been easier to delay school start times for a younger year group. Alternatively, feedback suggested that it may have been easier to involve sixth form students (16–19-year-olds), in particular to recruit sixth form colleges, educational institutions dedicated to this particular age group. Teachers believed that this group was likely to have more independence and a more flexible timetable with ‘free’ study periods and thus a greater ability to adopt a delayed start time.

### 4.7 Financial impact

The offer of £1000 to acknowledge the time and resources required to take part in the study was not financially sufficient for a school to be able to delay their start time, particularly with the existing economic pressures faced by schools. Although estimates are not directly transferable to the UK, the cost of delaying start times in a US school has been found to be much greater than the equivalent of £1000 [49]. This may not be the most critical concern for schools facing everyday financial realities, but wider and long-term societal implications could help influence schools. For example, a study using a modelling approach that focused on benefit-cost ratios of later school start times in 47 US states, weighed immediate costs occurred against potential economic benefits over time related to improved academic performance and decreased motor vehicle accidents. This RAND report suggested that the benefits of later start times to the economy would outweigh immediate costs [50].

### 4.8 Logistics/transport

It may have been beneficial prior to contacting schools to have investigated how students travelled to each particular school (eg, coaches organised by the school or public buses) so that a strategic plan could have been suggested offering ways to approach the issue of transportation. This could also have helped differentiate between transportation solutions for urban and rural schools. Similarly, a review including strategies for consideration when delaying school start times in the US, noted not only that transportation issues would vary by school district but also that it would be difficult to predict the issues that might arise without an evaluation of that district [51].

### 4.9 Alternative approach to research

Finally, an alternative, pragmatic approach to researching delayed school start times may be applicable in the UK. It could be more successful to establish which schools might be interested in delaying their start times, recruiting these interested schools to a future possible study, and then sourcing the funding required. Equally, a stepped-wedge design may be a more viable alternative to the classical RCT paradigm. All schools would receive the intervention but at different times, with each school providing baseline control data.

## 5 Conclusion

This study demonstrates the difficulty of recruiting schools to an RCT including a delayed start time as one of the interventions. Schools were interested in the topic of sleep and in educating students about how to get better sleep but were adamant in their view that being randomised to a change in start times was an unacceptable risk. Although an RCT is considered the ‘gold standard’ of research, the Teensleep study highlights the need for a pragmatic and alternative approach to evaluating the effect of school start times on sleep and other domains. The feedback received from schools during the course of recruitment could assist future studies by helping researchers to avoid the pitfalls encountered and to maximise the chances that schools will be willing to implement a change in start times.
